# In silico identification of phytochemical inhibitors for multidrug-resistant tuberculosis based on novel pharmacophore generation and molecular dynamics simulation studies

**DOI:** 10.1186/s13065-024-01182-7

**Published:** 2024-04-18

**Authors:** Bader S. Alotaibi

**Affiliations:** Department of Clinical Laboratory Sciences, College of Applied Medical Sciences, Shaqra University, Al- Quwayiyah, Riyadh, Saudi Arabia

**Keywords:** NPASS, SANCDB, Virtual screening, Molecular dynamics simulation, RpsA

## Abstract

**Background:**

Multidrug-resistant tuberculosis (particularly resistant to pyrazinoic acid) is a life-threatening chronic pulmonary disease. Running a marketed regime specifically targets the ribosomal protein subunit-1 (RpsA) and stops trans-translation in the non-mutant bacterium, responsible for the lysis of bacterial cells. However, in the strains of mutant bacteria, this regime has failed in curing TB and killing pathogens, which may only because of the ala438 deletion, which inhibit the binding of pyrazinoic acid to the RpsA active site. Therefore, such cases of tuberculosis need an immediate and effective regime.

**Objective:**

This study has tried to determine and design such chemotypes that are able to bind to the mutant RpsA protein.

**Methods:**

For these purposes, two phytochemical databases, i.e., NPASS and SANCDB, were virtually screened by a pharmacophore model using an online virtual screening server Pharmit.

**Results:**

The model of pharmacophore was developed using the potential inhibitor (zr115) for the mutant of RpsA. Pharmacophore-based virtual screening results into 154 hits from the NPASS database, and 22 hits from the SANCDB database. All the predicted hits were docked in the binding pocket of the mutant RpsA protein. Top-ranked five and two compounds were selected from the NPASS and SANCDB databases respectively. On the basis of binding energies and binding affinities of the compounds, three compounds were selected from the NPASS database and one from the SANCDB database. All compounds were found to be non-toxic and highly active against the mutant pathogen. To further validate the docking results and check the stability of hits, molecular dynamic simulation of three compounds were performed. The MD simulation results showed that all these finally selected compounds have stronger binding interactions, lesser deviation or fluctuations, with greater compactness compared to the reference compound.

**Conclusion:**

These findings indicate that these compounds could be effective inhibitors for mutant RpsA.

## Introduction

Tuberculosis (TB) is one of the most devastating and death-leading diseases worldwide, infecting a number of people every year. Mycobacterium is the main disease-causing pathogen infecting almost one-third of the world’s population. TB is mainly a lower respiratory disease, but it spreads throughout the body [1]. As per the World Health Organization report, both HIV and TB are highly lethal [2]. Because of the lack of properly developed treatment regimens, particularly against MDR (Multidrug-resistant) and EDR (Extensively drug-resistant) pathogens, tuberculosis is among the top 10 global causes of mortality [2]. Tuberculosis (TB) is usually treated with Pyrazinamide (PZA) in combination with other drugs like isoniazid, ethambutol, and rifampicin considered first-line regimens for tuberculosis [3]. PZA is an inactive drug that is converted into pyrazinoic acid (POA) the active form of PZA, inside the body of the pathogen by an enzyme pyrazinamidase coded as pncA [4, 5]. The POA can then binds to the cellular protein ribosomal Protein S1 coded as RpsA and Clpc1 for the pathogen, killing the bacterium by halting trans translation. However, the mycobacterium has developed resistance to this first-line regimen as well as to other allopathic drugs (chemicals), particularly against POA by different level mutations like deletion of alanine 438 from the alpha chain of ribosomal protein S1 RpsA [6]. Therefore in this study we have examined phytochemical (chemicals of plant origin) against these mutated strains which are the unique approach of this work (Comment#1). Drug resistance to mycobacterium makes the disease a serious risk to global health security and has a dangerous impact on public health in both middle and high-income nations [6].

RpsA is an important target for POA, as reported in 2011 [7]. It has also been shown that POA checked the formation of RpsA-tmRNA complex by binding to wild-type RpsA at C terminal or M. Smegmatis but cannot bind to the alanine 438 deleted mutant of *mycobacterial* RpsA (RpsA 438 A) [7]. By this deletion, the pathogens developed resistance to POA, and the binding site of the receptor changed; hence the POA could not bind, and the bacterium survived. RpsA has a key role in the initiation of mRNA translation, particularly trans-translation through the ribosome-sparing process with rare codon presence within *Mycobacterium* [8, 9]. RpsA protein is composed of four S1 domains. The C terminal domain is formed of a 4th domain, and the mutation occurs in this C terminal domain [10, 11]. Therefore, it is required to have a drug to bind to mutated RpsA and cure TB, with fewer or no side effects and permissible ADMET properties, such regime may be better obtained by targeting pathogen with phytochemicals. In the recent a paper has been published which have suggested a ligand named zrl15 with a better impact against mutated RpsA, and their result has been explored in silico as well as in vitro by different techniques [12]. Previously we have sought out leading compounds for such mutated strains by virtually screening of chemical libraries like ChemBridge and Zinc databases which have prominent binding to mutated proteins [13], here we have screened phytochemical databases i.e., NPASS and SANCDB by using an online server Pharmit [14]. By searching these phytochemical databases, we have found a number of lead compounds through virtual screening via an online server, Pharmit (http://pharmit.csb.pitt.edu).

## Methods

### RpsA^WT^, RpsA^del438A^ and Ligand (POA), zrl15 interaction study

First of all, complex X-ray crystallographic structure of RpsA was download from the protein data bank with PDB ID 4NNI [15]. The interactions of this wild type were studied using a molecular interaction protocol implemented in MOE. Then RpsA mutant structure was developed by deleting the residue alanine 438 in Pymol software and in the same way the molecular interaction was studied. The reference ligand (zrl15) was then docked in the binding pocket of mutated RpsA on the basis of already bound POA via MOE after minimization. Hydrogen atoms were added and the residue selenomethionines was replaced by methionine in MOE. The key residues for substrate binding found were Lys303, Phe307, F310, and Arg357 [11].

### Virtual screening on the basis of pharmacophore model

In computational drug development, pharmacophore-based virtual screening is one of the most vital steps to search large libraries to seek out LEADS against a specific pathogen. There are number of software and online servers to do the job like Dock Blaster [16], iDrug [17], iStar [18], e-LEA3D [19], and MTiOpenScreen [20], which give results in hours or days with the limitation to screen the chemical databases of small size only. On the other hand, Pharmit is an online server with the algorithm that can screen compounds libraries on the basis of pharmacophore model or molecular shape and can rank the results by energy minimization [14]. By using Pharmit large databases of compounds could be screened on the basis of pharmacophoric features or molecular shape. Here in this study, we have screened two phytochemical databases with the reference compound zrl15 via Pharmit. The phytochemical databases were used mainly under the criteria that this study seeking regime of MDR/EDR cases through phytochemicals. 154 hits were retrieved from NPASS database and 22 hits were retrieved from SANCDB database based on the pharmacophore with features like two hydrogen bond donors, i.e., nitrogen 11 and nitrogen 12, two hydrogen bond acceptors i.e., oxygen 3 and 13 and two hydrophobic i.e., Sulphur 6 and Carbon14 on the basis of molecular shape of zrl15, followed by energy minimization of these hits and got their most stable conformers. The energy minimized hits were then docked in the binding pocket of mutated RpsA in MOE.

### Docking

The docking was performed for the prediction of binding modes and lead compounds selection from the initial hits, for this purpose all initial hits were docked in the binding pocket of mutated RpsA using MOE-Dock v2016. A total 5 conformations for each compound were generated using the default MOE parameters, i.e., placement: Triangle Matcher, Rescoring: London dG, GBVI/WSA dG, and Refinement: Rigid Receptor. Our docking protocol has been found reliable by testing it via redocking protocol of MOE using the SVL script. The RMSD between the co-crystalized ligand and re-docked conformation was observed to be 0.78 Å, which is within the allotted reliable range of docking. Based on docking score and molecular interaction analysis, the top 03 compounds were subjected for further assessment. Finally, the selected 03 compounds were subjected to molecular dynamics simulations.

### Binding affinities and binding energy calculations

For all the four complexes binding affinities and binding energies were calculated using GB/VI (Generalized Born / volume integral) built in MOE to find the most active ligand against the bacterium and Lipinski rule of five were applied.

### Drug likeness and ADMET (absorption, distribution, metabolism, excretion and toxicity) properties of finally selected compounds

Ordinary process of discovery and development of drug designing is in endanger in term of economy which is usually faces to unexpected even worst failures in various stages of drug development and discovery. The main reason for these failures may usually the efficacy and safety limitations which are principally concerned to these pharmacokinetics properties like absorption, distribution, metabolism, excretion (ADME) properties and different toxicities (T). Therefore, ADMET analysis is of prime importance to perform in the process of drug discovery and development. Here we also performed these analyses using free, online pkCSM server available at http://biosig.unimelb.edu.au/pkcsm/prediction.

### Molecular dynamics (MD) simulations

All finally retrieved/selected compounds were subjected to all atom’s MD simulations. MD simulation combines different techniques based on highly advanced and complex algorithm, which essentially giving deep and necessary molecular interaction information *in silico*. In drug design and discovery, it is of utmost importance to study these interactions. In this study, we have carried out all necessary MD simulation analysis like RMSD, RMSF, RoG, DCCM, and PCA for the finally selected compounds using AMBER v2018 with the force field (ff14SB). The ff14SB protein force field was employed for the protein while GAFF was used as the ligand force field. For the solvation of each system, the tip3p water model with box dimension 8.0 °A was used. The MDS was performed by incorporating the isothermal isobaric ensemble (NPT) at 310 K [21, 22]. The system was neutralized and solvated using counter ions (Na + and Cl−) via LEAP module and octahedral box of TIP3P water model with a 12.0 Å buffer was used respectively. The cutoff distance 10 Å was used to determine the van der Waals and long-range electrostatic interactions was determined using the Particle Mesh Ewald PME algorithm was used [22]. For the constraining of the bonds involving hydrogen atoms, SHAKE algorithm was used, with 0.5 ns of constant pressure equilibration at 300 K [23]. The temperature was control by Langevin dynamics [24]. Finally, MD simulation of 200ns was carried out for all equilibrated complex systems at constant temperature and pressure [25]. MD trajectories were analyzed by using CPPTRAJ module of Amber v2018.

#### Dynamic cross-correlation map

Dynamic Cross-Correlation Map analysis was performed for all complexes in order to examine the comparison of Cα atoms throughout the correlation matrix [26]. On the basis of Cα carbon atoms, the DCCM analysis used 9800 snapshots. DCCM was analyze by Cpptraj and the data was plot by the Origin software [27]. In DCCM graph, there are two types of correlations, positive correlation and negative correlation. A positive correlation shows that the movement of protein and ligand occurs in the same direction and the complex achieves stability while interacting with each other. On the other hand, the negative correlation shows that the ligand moves away from the binding pocket and gives instability. In DCCM map the color intensity represent the strength of the positive and negative correlations. The dark red to light red and darker indigo to light indigo to yellowish green color indicate the positive and negative correlations. The positive correlation is indicated by red color whereas the negative correlation is indicated by indigo color: The more color intensity represents good respective correlation and vice versa.

#### Principal component analysis

Another important analysis carried out for the finally selected compounds were Principal Component Analyses. The analysis was assessed by using cpptraj package [28]. The calculation of covariance matrix was performed based on coordinates of Cα and eigenvalues and eigenvectors analysis were calculated with matrix of diagonalized covariance. The atoms (ligand and receptor) direction of movement is indicated by eigenvector (Pc), whereas the mean square fluctuations of the atoms of the complex is indicated by corresponding eigenvalues. For the calculation and plotting purposes PC1 and PC2 were used to check their mobility.

#### **Binding free energy** MM/GBSA and MM/PBSA **calculation**

MM/GBSA and MM/PBSA are two widely used techniques for the calculation of the free energy of binding. The binding free energy (BFE) of the systems was calculated using the MMPBSA.PY script [29]. To calculate BEF, the last 500 snapshot samples were used. We used the MMPBSA and MMGBSA methods to compute the binding free energy of protein-ligand complexes [30]. For the binding free energy calculation, the following equation was used.

∆𝐺𝑏𝑖𝑛𝑑 = ∆𝐺𝑐𝑜𝑚𝑝𝑙𝑒𝑥 − [∆𝐺𝑟𝑒𝑐𝑒𝑝𝑡𝑜𝑟 + ∆𝐺𝑙𝑖𝑔𝑎𝑛𝑑].

Here, the term ∆Gbind denotes the total binding energy, and the other terms in the equation denote the free energies of the complex, receptor, and ligand.

## Results and discussion

### RpsA^WT^, RpsA^del438A^ and Ligand (POA), zrl15 interaction study

**Fig. 1:**
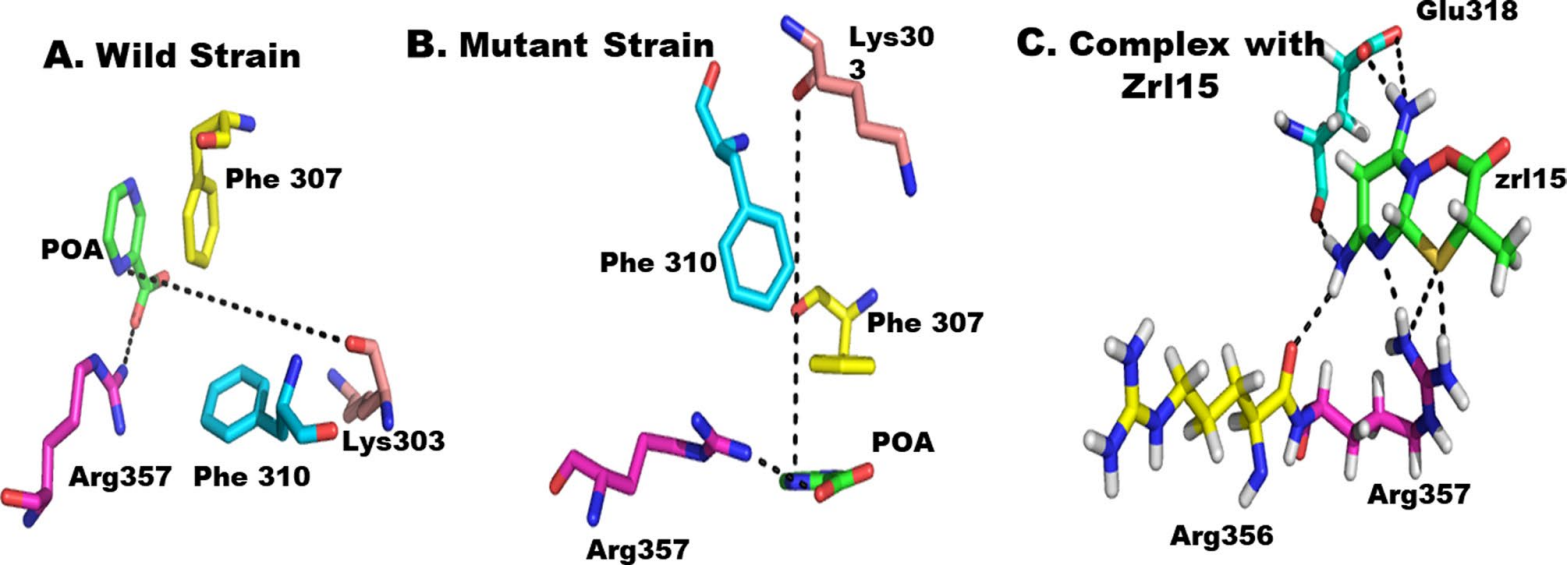
In mutant stains, the Phe 307 and Phe 310 are too far from POA to form π- π interactions

Molecular interaction studies of all three complexes i.e., RpsA^wT^ complex with POA, RpsA^del438A^ complex with POA and RpsA^del438A^ complex with zrl15 were studied by docking all three inhibitors in the binding pocket of mutant RpsA in MOE prior to pharmacophore based virtual screening. It has been found that RpsA^wT^ and RpsA^del438A^ complex with zrl15complex were having much stronger interactions as compared to RpsA^del438A^ complex with POA (Fig. 1**)**. Therefore, on the basis of RpsAdel438A, zrl15 complex pharmacophore based virtual screening was carried out.

### Pharmacophore-based database screening

**Fig. 2:**
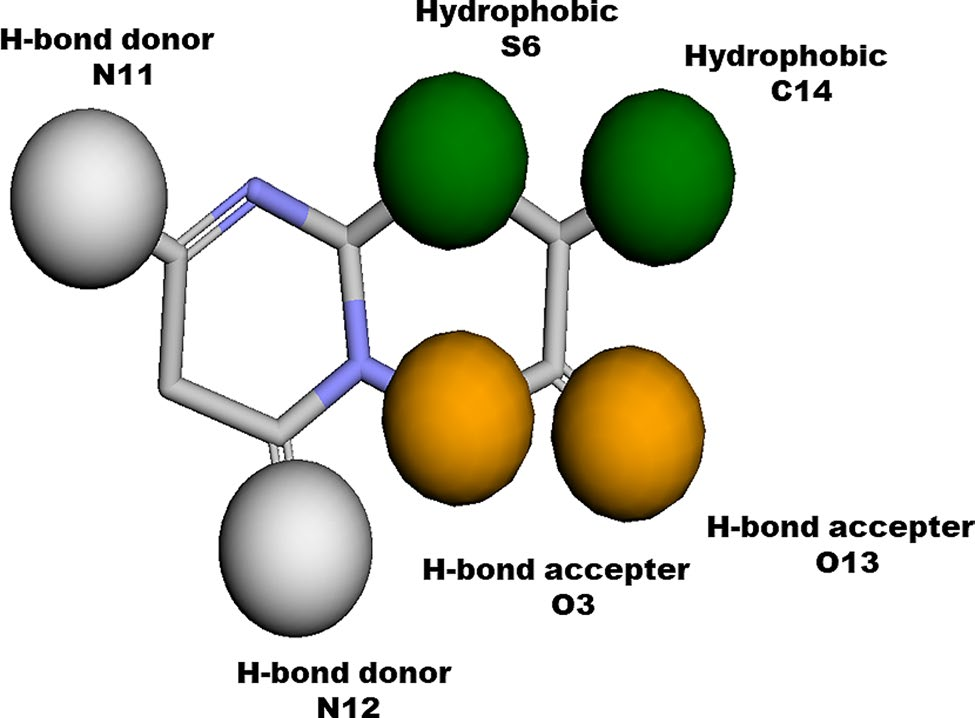
Typical pharmacophores generated on the basis of zrl15 via Pharmit. The model contains six features. The hydrogen bond donor features are colored *grey*, the hydrogen bond acceptor features are colored *orange* and hydrophobic features are colored *dark green*

Two phytochemical databases were virtually screened with the reference compound zrl15 by an online server Pharmit. Pharmit is an online tool that performs pharmacophore based virtually screening. Pharmacophore was generated on the basis of zrl15 with a total of six features. Two features were Hydrogen bond donor, i.e., Nitrogen 11 and 12, two were Hydrogen bond acceptor, i.e., Oxygen 3 and 13, and two were hydrophobic, i.e., Sulphur 6 and Carbon14 as shown in Fig. 2. NPASS and SANCDB databases were virtually screened with the generated pharmacophores. 22 hits were retrieved from SANCDB database and 154 hits were retrieved from NPASS database. Energy minimizations of all these hits were carried out in complex with RpsA. The energy minimized hits were subjected to docking in the binding pocket of RpsA for intermolecular interaction studies.

### Molecular docking

**Fig. 3:**
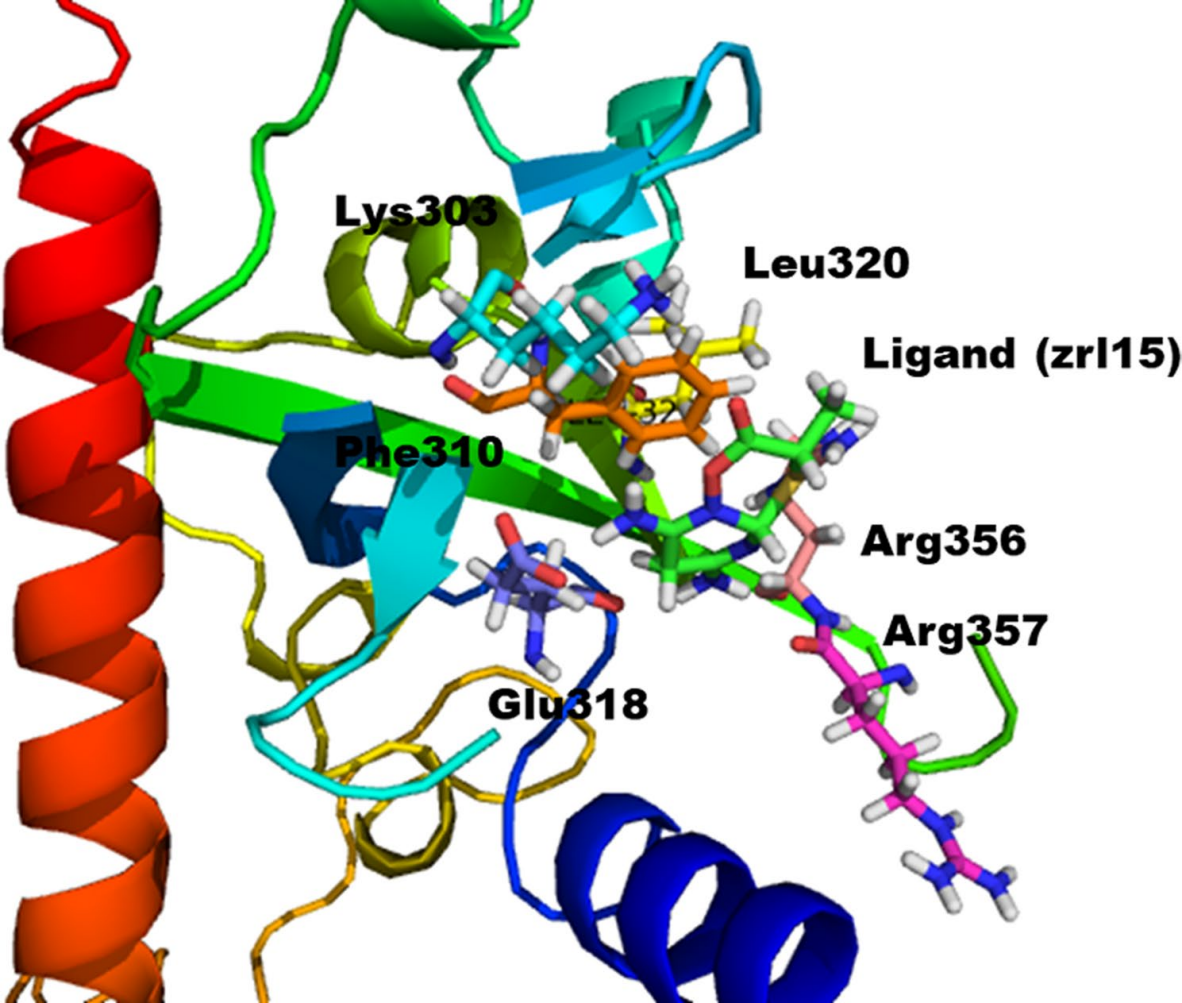
Binding pocket of the protein

Hits of both databases were docked in the binding pocket of mutant RpsA using the MOE-Dock. For each hit five conformations were generated. The top 4 compounds were selected among all docked compounds on the basis of having the best docking scores and maximum molecular interactions with active site residues like Phe 310, Arg 357, Lys 303, Glu 318, Arg 356, Leu 320, His 322 and Phe 307 from both databases as shown in binding pocket of protein Fig. 3. Their docking scores are given in Table 1. For further analysis, the binding energy and binding affinity of the top 4 compounds were calculated, and then the top 3 compounds were subjected to molecular dynamics simulations.

### Binding affinity and the calculation of binding energy

The binding energies and binding affinities of the docking complexes of finally selected 4 compounds were calculated using the generalized born / volume integral (GB / VI) implemented in MOE to find the most active ligand and Lipinski rule of five were applied. All the four hits were following Lipinski role of five as shown in Table 1. They have been found non-toxic with much higher stability or lower binding energies as shown in Table 1.

**Table 1:** Docking scores, binding affinity and binding energy along with Lipinski rule of five values of top hits and reference compounds (Deviation from the rules in some values may only because of their phytochemical nature as most of the phytochemicals are successful marketed drugs but do not follow the Lipinski Role of Five)

Compound ID	Docking Scores	Binding Affinities (kcal/mol)	Binding Energies (kcal/mol)	Lipinski Role of Five			
				H-bond donor	H-bond acceptor	Log P	Molecular Weight (g/mol)
NPC6836	−5.3173	−7.01	−49.04	7	12	−2.35	448.42
NPC227485	−4.5465	−5.92	−42.94	9	11	−0.69	424.36
NPC227980	−5.4021	−6.81	−47.63	6	12	−1.80	446.40
SANC00619	−4.8770	−6.79	−45.40	02	08	7.52	548.76
Zrl15	−4.518	−4.96	−28.40	2	2	−0.80	214.24

### Pharmacokinetics properties

Pharmacokinetics (ADMET) properties have been performed last selected compounds along with reference compounds. These compounds have been found of obeying drug likeness like lipinski rules of five as well having allotted range of ADMET properties. According to lipinski rules of five “a drug like compound must not have hydrogen bond acceptor more than 10, hydrogen bond donor not more than 5, water octanol coefficient not more than 10 and molecular weight must be less than 500 Daltons”. Their ADMET properties have shown Table 2.

**Table 2:** Pharmacokinetic (ADMET) properties

Category	Property with Unit	Compound’s ID				
		NPC6836	NPC227980	NPC227485	Zrl15	
Absorption	Water solubility (log mol/L)	−1.971	−2.726	−4.748	−1.563	
	Intestinal absorption (%)	19.146	30.207	99.683	67.684	
	Skin permeability(log Kp)	−2.735	-2.735	−2.735	−3.28	
Distribution	VDss (human) (log L/kg)	0.186	-0.245	−0.658	−0.133	
	BBB permeability	−1.399	-1.386	1.398	−0.664	
	CNS permeability (log PS)	−5.669	-5.675	−1.078	−3.55	
Metabolism	CYP2D6 substrate	No	No	No	No	
	CYP3A4 substrate	No	No	No	No	
	CYP1A2 inhibitor	No	No	No	No	
	CYP2C19 inhibitor	No	No	No	No	
	CYP2C9 inhibitor	No	No	No	No	
Excretion	Total Clearance(log ml/min/kg)	0.767	1.14	0.749	0.497	
	Renal OCT2 substrate	No	No	No	No	
Toxicity	AMES toxicity	No	No	No	No	
	Max. tolerated dose (human) (log mg/kg/day)	−0.043	0.046	2.647	0.897	
	Oral Rat Acute Toxicity (LD50) (mol/kg)	2.977	3.405	−0.476	2.414	
	Hepatotoxicity	No	No	No	Yes	
	Skin Sensitization	No	No	No	No	

### Molecular dynamics simulations

**Fig. 4:**
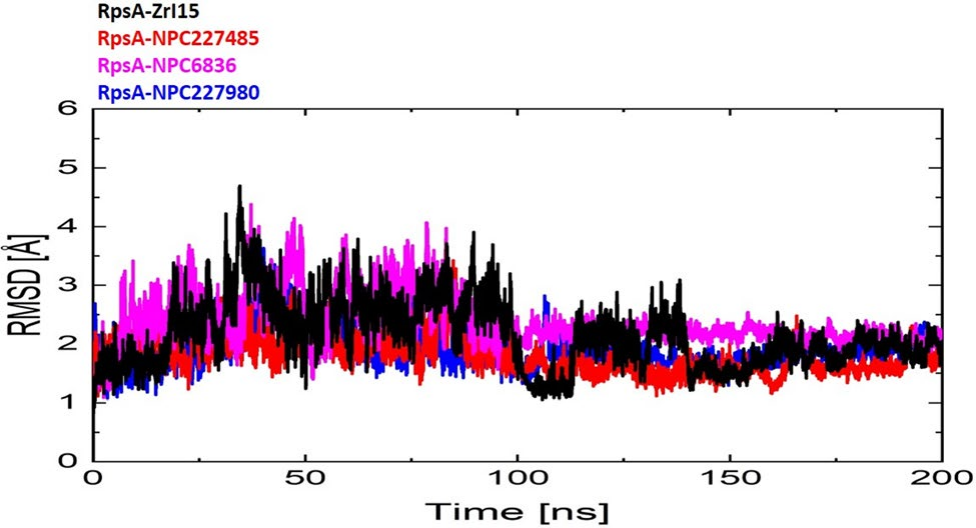
RMSD Analysis in clubbed of the finally selected compounds

Molecular dynamics (MD) simulations of all 03 finally selected compounds in comparison with the reference compound were performed. Various Molecular dynamics simulations analysis were performed like RMSD, RMSF, RoG, DCCM, and PCA, and their results found with better those better dynamics behavior compared to the reference compound. The stability of the complexes was studied considering the Root Mean Square Deviation (RMSD) analysis. 200 ns simulations were run to study the interactions and stability of these complexes. From RMSD curve, it has been found that the reference compound was having a little bit more deviation compared to the selected compounds (Fig. 4**)**. The RMSD curve of reference compound goes beyond the 4 Å even touching 5 Å around 30-40ns shows more instability of the complex compared to selected compounds. At start of the trajectory, the deviation of compounds NPC227485 and NPC227980 seems parallel, but beyond 30–50 ns there is a greater deviation in the reference compound from the alpha carbons of protein. The greater deviation of the reference compound has been seen throughout the trajectory compared to these two compounds, in the case of NPC6836, the reference compound and NPC6836 both have comparable deviation but still NPC6836 seems with greater stability because of more deviation of reference compound at 30–40 ns as shown in Fig. 4.

**Fig. 5:**
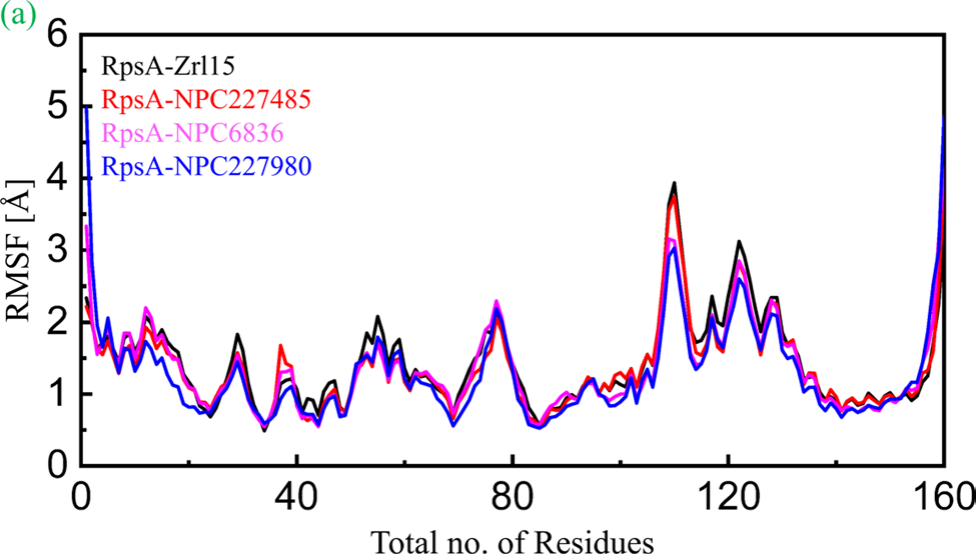
RMSF analysis of the finally selected compounds

To get more information about the impact of the deletion of ala438 residue, Root Mean Square fluctuation (RMSF) analysis were also performed. In this analysis fluctuation of each residue was calculated by RMSF analysis. It has been found that the reference compound showed more fluctuation as compared to any selected compound, particularly at residues 110–120 as shown in Fig. 5 goes beyond the 4 Å at this point. Although the compound NPC227485 has also shown greater fluctuation at the same point but still seems better than the reference compound throughout the trajectory. The compound NPC227980 has shown much higher fluctuations at the start and end of the trajectory but still in the remaining residue sequence it has shown lesser deviation as compared to zrl15. The third compound NPC6836 seems much better compared to all others as it has uniform fluctuations throughout the 100 ns simulations. RMSF analysis has been illustrated in Fig. 5.

**Fig. 6:**
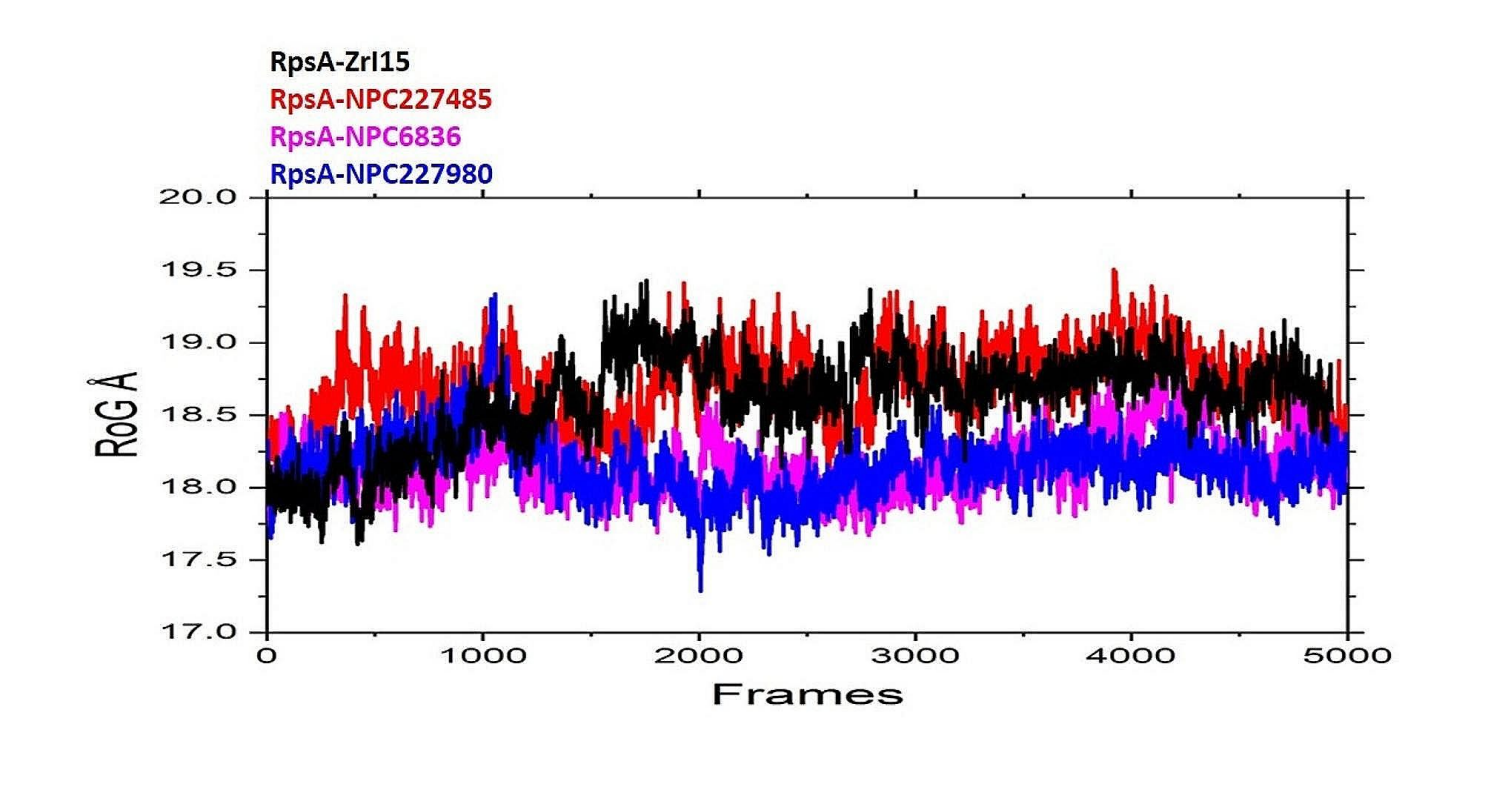
Radius of gyration of the finally selected compounds

In this work, we computed the radius of gyration of a protein molecule from its spatial structure, taking atoms as balls of the same radius 1.5 Å and the same mass along the 200 ns simulation trajectories. The radius of gyration was computed for a preset size range, with the average size of protein-ligand complex size being approximately the same in each structural class. Finally, our results showed that throughout the simulations the folding process of RpsA complex with different attached inhibitors like NPC6836, NPC227485, and NPC227980 is characterized by somewhat similar behavior of the radius of gyration, with no significant increase of the native residues contacts and of secondary structure content of inhibitors; however in later part of trajectory reference compound zrl15 have shown increased radius of gyration in comparison to the selected inhibitors specifically NPC6836, NPC227980 as shown in Fig. 6. In the last part of the simulation the radius of gyration is almost constant, whereas the native contacts percentage and the secondary structure content increase in an almost concerted way. This folding path is in agreement with the molecular docking suggestions and results.

#### Dynamics Cross correlation map analysis

**Fig. 7:**
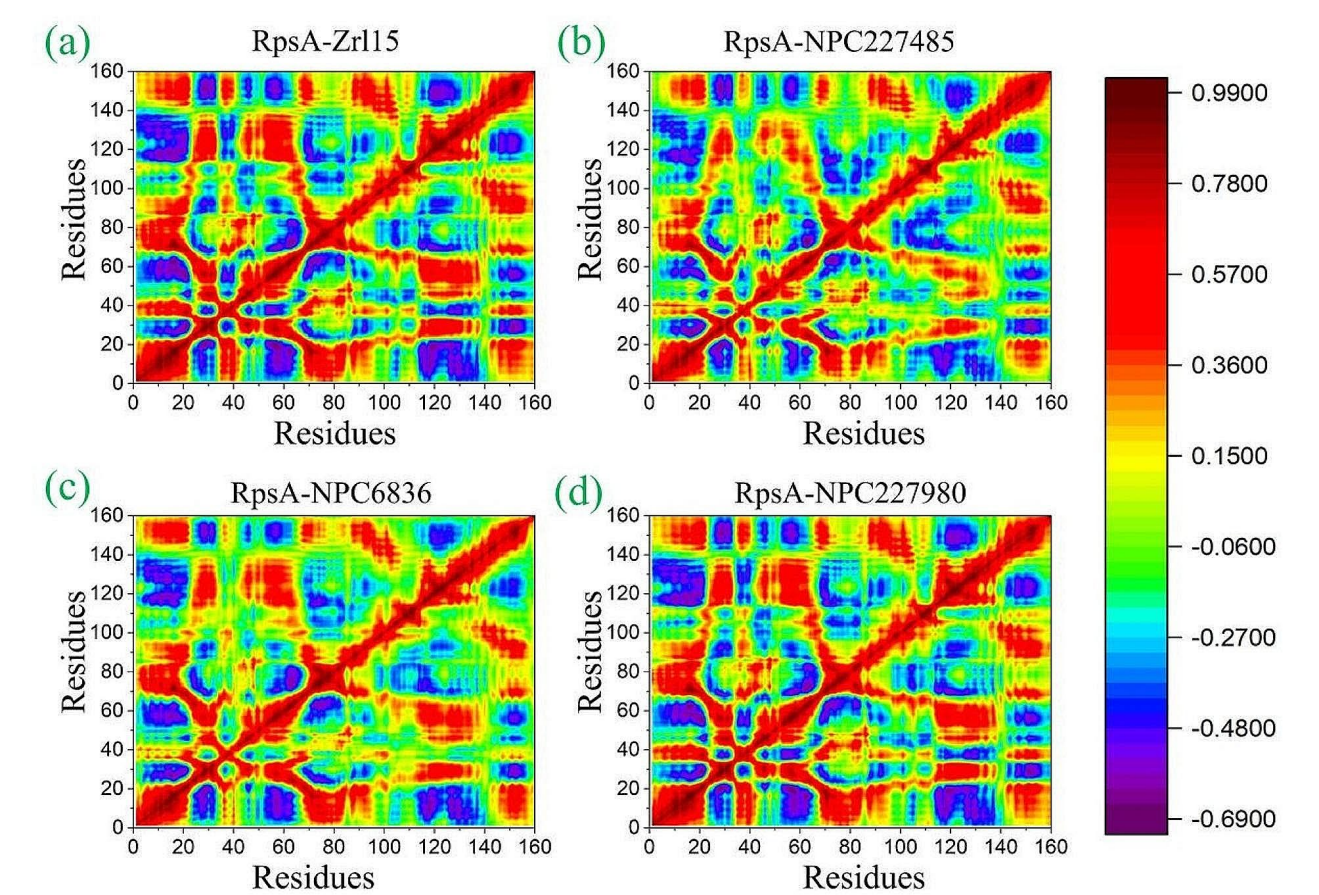
Dynamics Cross Correlation Map of Finally selected compounds

To explore the functional displacement of all system as a function of time the DCCM analysis was performed. The results of the analysis show that all compounds have about similar correlations including reference compounds. All compounds have shown strong positive correlations as shown in Fig. 7. This further strengthen that the positive correlation might be due to the observed interactions of these compounds with the binding site residues (residue regions 40 − 80, 120, and 160). Overall, the Dynamics Cross Correlations Map graphs showed All compounds have shown stronger positive correlation, specifically at the positions where compounds mostly interact with residues, i.e., active site residues and some additional nearby residues (Fig. 7**)**. Among the residues the dark indigo color represents strong negative correlation whereas the dark red color represents a strong positive correlation. The residues that are positively correlated move in the same direction whereas those residues that are negatively correlated move in the opposite direction. The interactions details of all compounds and residues are shown in Table 2.

#### Principal component analysis of RpsA mutant complexes

**Fig. 8:**
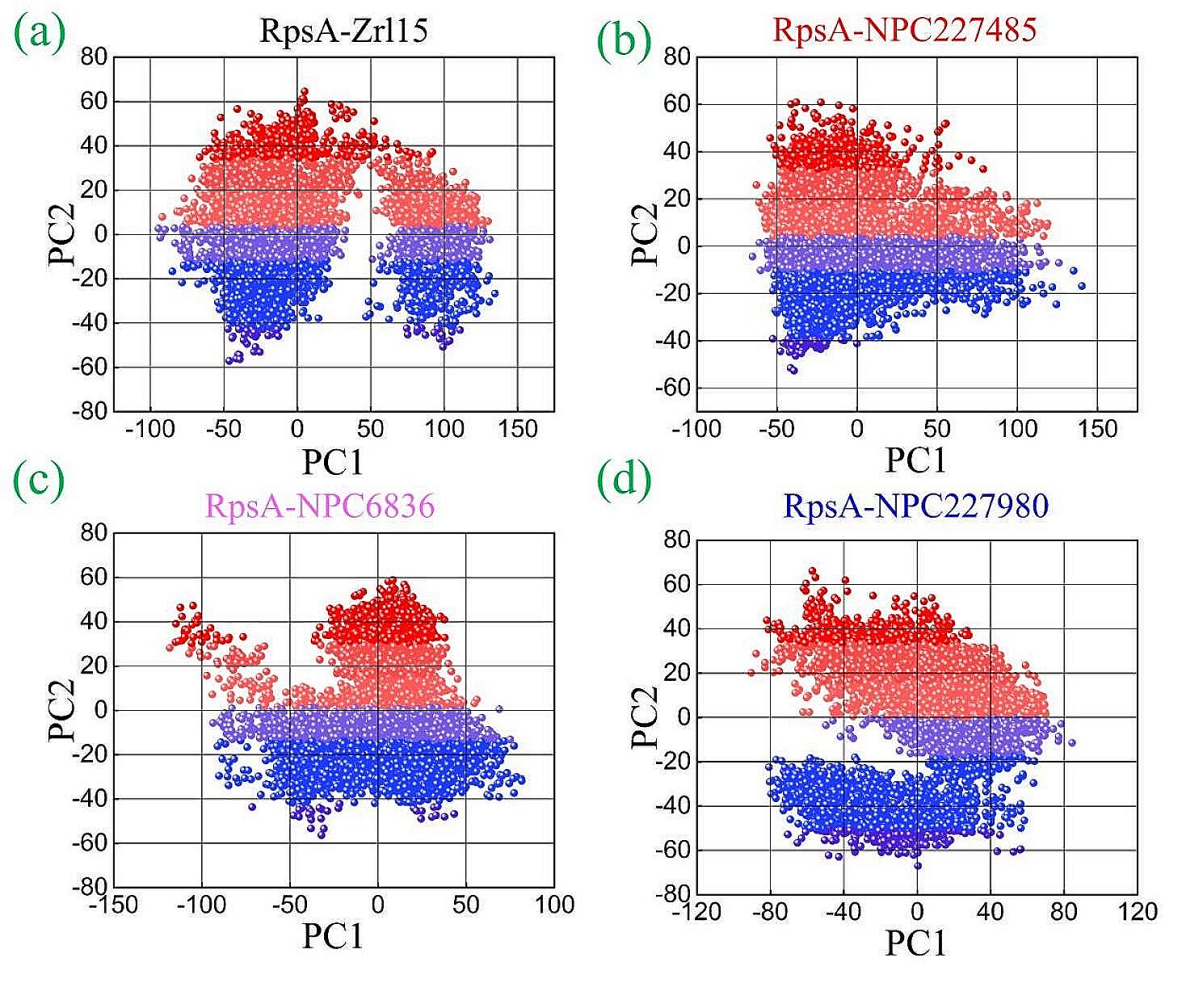
Principal component analysis of the finally selected complexes

The dynamically favorable conformational changes of compounds and protein were explored by principal component analyses of mutant complexes. The 100 ns dynamics simulation results show that all 3 complexes along with the reference compounds have comparably similar patterns. The deviations across the different energy states were found in different patterns but still it was quite comparable in all complexes. It has been shown that the compounds as well as resides in the same site during the whole MD simulation resulting in similar energy states as shown in Fig. 8. With the applied eigenvalues and eigenvectors, the most dominant structural interactions of the complexes were explored. It has been found that all complexes have exhibited a motion in different direction from one another. It was observed that the reference compound’s phase motion was mixed and clustered (Fig. 8**)**. In the case of NPC227485 are highly arranged and compact compared to other complexes, covering the area of − 75 to + 125 along the PC1 and − 60 to + 60 along the PC2, such arranged and compact motion of this complex indicates good magnitude/stability of the complex. The other two complexes, i.e., NPC6836 and NPC227980, have also been found with more arranged and compact dots (motions) as compared to the reference complex as shown in Fig. 8. Shortly, the overall results of the analysis showed that the selected compounds were more arranged and compact as compared to the reference compound.

#### Binding free energy calculation MMGBSA and MMPBSA analysis

The MMGBSA and MMPBSA analysis were carried out for all the protein-ligand complexes and the result was compared with the control complex. The binding energy calculation revealed that among all the systems the RpsA-NPC227980 complex revealed a strong binding toward the receptor as compared to all other systems as confirmed by MMPSA and MMGBSA analysis. Table 3 represents the results of MMPBSA analysis and Table 4 represents the result of MMGBSA analysis for all the systems.

**Table 3:** MMPBSA analyses for the complexes

Complex	VDWAALS	EEL	ENPOLAR	EPB	DELTA TOTAL kcal/mole
RpsA-NPC227485	−26.6921	−29.9760	−21.2434	30.1411	−9.4329
RpsA-NPC6836	−31.3798	0.3502	−19.4179	8.7654	−7.1571
RpsA-NPC227980	−42.7893	−5.4652	−24.5870	18.8990	−10.1298
RpsA-ZrI15	−66.4469	−4.8192	34.8145	34.8145	−5.0666

**Table 4:** MMGBSA analysis for all the complexes

Complex	VDWAALS	EEL	ESURF	EGB	DELTA TOTAL kcal/mole
RpsA-NPC227485	−32.8640	−15.0445	−3.9041	29.2027	−22.6100
RpsA-NPC6836	−24.2607	−4.1676	−2.2783	10.1116	−20.5950
RpsA-NPC227980	−28.8158	−5.3400	−3.5226	14.3454	−23.3331
RpsA-ZrI15	−19.6302	−2.6766	−2.5421	12.8009	−12.0480

### Protein ligand interaction profile of the finally selected complexes

**Fig. 9:**
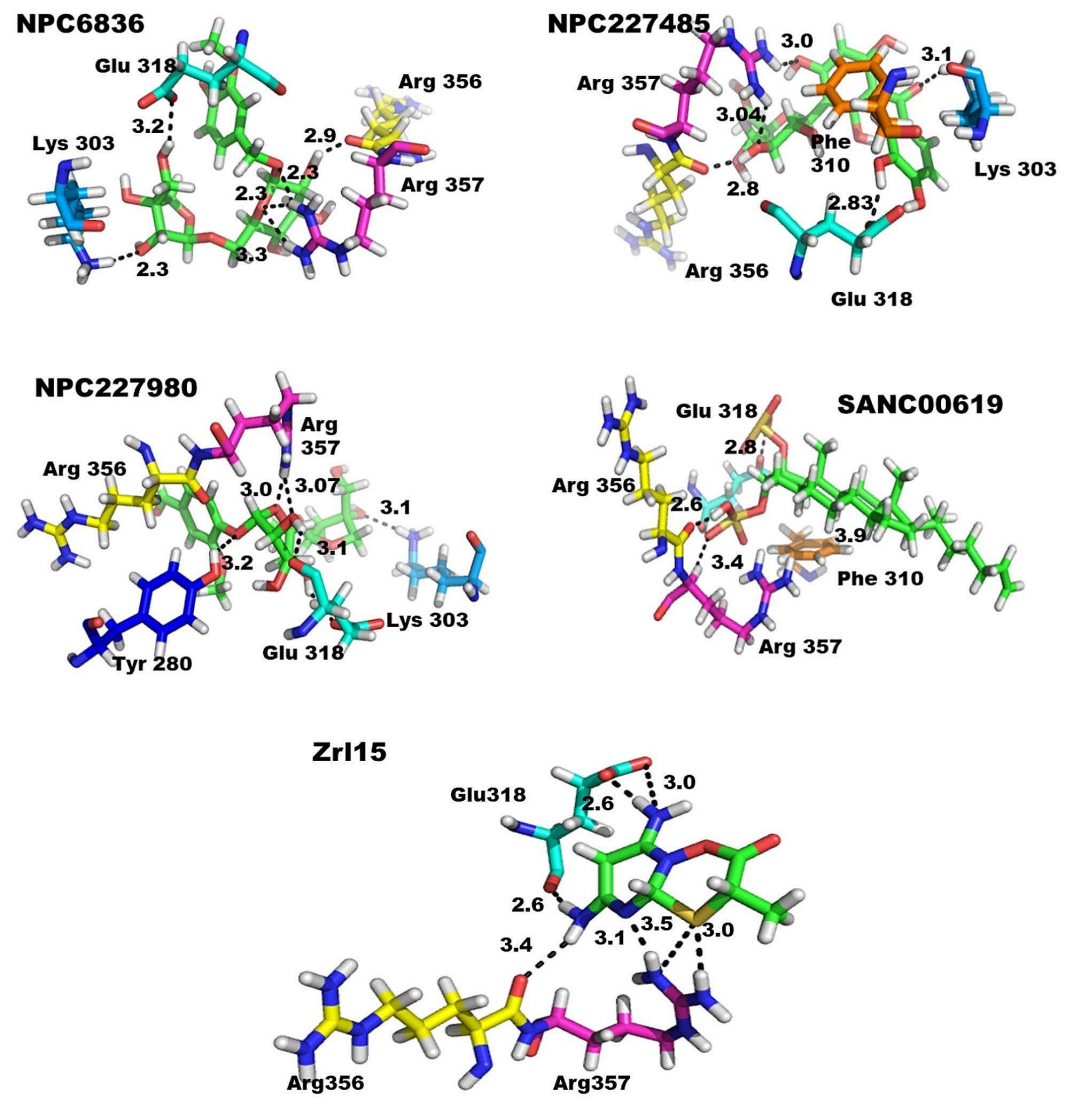
Protein Ligand interaction with interaction distances of finally selected compounds, the green color shows the ligand in the entire compound

Protein ligand interaction profile showed the exact interactions of the ligand and the protein, which is also referred as protein-ligand interaction visualization. The protein-ligand interaction was visualized using Pymol. All selected compounds were found in stronger interaction in the protein active sites compared to the reference compound. The only compound with a little bit poor interaction was SANC00619 which has shown comparable interaction with the reference compound as shown in Fig. 9 and the interaction detail is given in Table 5. All remaining three compounds were having quite stronger and stable interactions as shown in Fig. 7 and the detail is given in Table 5. The compound NPC6836 has extra-ordinary binding interaction, it has formed six strong hydrogen bonds with mutant RpsA. The oxygen 18 of the compound bonded to nitrogen of Lys 303, oxygen 21 bonded via hydrogen bond to Glu 318, oxygen 25 of the ligand bonded to oxygen of Arg 356, similarly oxygen 7 of the compound bonded to the amino group of Arg 357, and oxygen 9 forms double hydrogen bonds to the amino groups of Arg 357 as given in Table 5. Among the other two compounds, NPC227485 also forms a number of hydrogen bonds and pi interaction as given in Table 5 and shown in Fig. 9, the compound NPC227980 also forms four hydrogen bonds and a single pi bond, Oxygen 21 forms a polar interaction with oxygen atom of Glu 318, the atom 23 (oxygen) interacts with the amino group (Nitrogen) of Lys 303 via polar bonds, the Oxygen 13 forms hydrogen bond with the Nitrogen of Arg 357 and Oxygen 21 and Oxygen 29 interact with O and OE2 of Glu 318 as shown in Fig. 9; Table 5.

**Table 5:** The details of Protein − Ligand Interaction (PLI) of the complexes of finally Selected Compounds and RpsA Protein

S.no	Compound ID	Ligand	Receptor	Interaction	Distance	Energy (Kcal/mol)
1.	NPC6836	O 21 O 25 O 7 O 9 O 9 O 18	OE2 Glu 318 O Arg 356 NH1 Arg 357 NH1 Arg 357 NH2 Arg 357 NZ Lys 303	H-donor H-donor H-acceptor H-acceptor H-acceptor H-acceptor	3.19 2.87 3.17 3.27 3.34 3.11	-1.0 -1.4 -1.5 -1.4 -1.1 -2.1
2.	NPC227485	O 10 O 28 O 29 O 10 O 21 C 27	O Arg 356 OE1 Glu 318 OE2 Glu 318 NH1 Arg 357 NZ Lys 303 6-ring Phe 310	H-donor H-donor H-donor H-acceptor H-acceptor H-pi	2.88 2.83 3.00 3.04 3.11 3.79	-1.7 − 3.0 -3.5 -0.8 -0.7 -0.5
3.	NPC227980	O 29 O 31 O 13 O 23 6-ring	O Glu 318 OE2 Glu 318 NH1 Arg 357 NZ Lys 303 CB Arg 356	H-donor H-donor H-acceptor H-acceptor Pi-H	3.10 3.24 3.07 3.17 4.52	-0.6 -1.1 -3.0 -1.0 -0.6
4.	SANC00619	O 29 O 35 O 33 C 23	OE2 Glu 318 O Arg 356 CA Arg 357 6-ring Phe 310	H-donor H-donor H-acceptor H-pi	2.85 2.66 3.41 3.91	-12.2 -8.0 -0.6 -0.5
5.	Zrl15	N 11 N 12 N 11 S6 S6	O Glu318 OE2 Glu 318 O Arg 356 NH1 NH2	H-donor H-donor H-donor H-acceptor H-acceptor	3.08 2.68 3.49 3.58 3.19	-2.2 -4.9 -0.9 -1.5 -3.6

### Discussions

As there is no proper treatment for MDR/EDR cases of TB (1) and there is an intense need of proper regimen which have high efficacy, allotted pharmacokinetics (ADMET) properties with fewer side effects. Scientist have done various effective rather somewhat successful efforts in this regard and found better antagonist for such cases like zrl15 (1) which has proven better both in in silico and in vitro activities. Likewise a number of effective chemicals found in another research published recently having even much better properties than zrl15 [13]. However all these efforts are still in the various stages of drug designing and development and there are no effective marketed drugs for such cases, secondly all these running drugs are of allopathic origin and may have little or more side effects on patients’ health. Here in this study we have carried out the same attempt for targeting the pathogen with some phytochemicals which have generally fewer side effects as compared to synthesized chemicals also conferring novelty to this work. Although the study is only confined to computational analyses but still we have found number of phytochemicals like NPC6836, NPC227980 and NPC227485 with better ADMET and other drug-likeness properties with no toxicity as shown in the Table 2. They have also been found much effective in terms of molecular interactions and docking scores as well as throughout the molecular dynamics simulations trajectories like have fewer deviation and fluctuations with the alpha carbons of targeted protein after binding as shown in Figs. 4 and 5, and 6. Based on these and other analyses like DCCM and PCA these selected compounds of plant origin may prove much effective against such cases of TB.

### Conclusions

Different researches have been carried out, and numbers of drugs have been sought out for the sake to have a short and effective treatment of M. tuberculosis. This study has also tried to determine a lead compound that has better efficacy and reduce the time period of treatment of the disease. Based on reference compound, three structurally diverse compounds were computationally identified. The different drug like properties of the identified compounds and reference compound were performed and compared by various *in silico* techniques. The results showed that the identified compounds were of equal importance as all of them were non-toxic and highly active against the targeted protein compared to zrl15. They were having strong molecular interactions with active site residues with good binding energy and binding affinity. They were having lesser deviations or fluctuations and highly compact assembly of the protein. On the basis of these results, they could be possible inhibitors for the mutant RpsA.

## Data Availability

No dataset is generated and/or analyzed that required submission to any repository.
